# The Choice of a Profession

**Published:** 1898-09-03

**Authors:** 


### The Hospital

A Journal of

The Medical Sciences and Hospital Administration.

Vol. XXIV.—No. 623.] Edited by Sir Henry Burdett, K.C.B., and Solomon C. Smith, M.D., M.R.C.P. [Sept. 3, 1898.

### THE CHOICE OF A PROFESSION.

Many causes have conspired to cast a sort of glamour over medicine as a profession, and thus to render it attractive to young people who shrink from what they look upon as more commercial methods of earning a living. The fact that medicine, if not in itself a science, is at least a scientific pursuit, that the practice of medicine is at every point in touch with charity, that medical practitioners not only are appealed to in sickness by the highest in the land, but are known to be on terms of friendship with the great ones of the earth, and, lastly, the honours and the titles and the wealth which fall to the lot of some at least of those who embrace medicine as a profession, all prove attractive to young people at that period of their lives when the necessity of providing themselves with their daily bread has never been urgently forced upon them, and when all that has to do with "filthy lucre," as they are in the school books taught to call it, seems mean, degrading, and to be avoided at any cost. We need hardly wonder then that as each session comes round again we find the lecture theatres filled with eager neophytes, ready, and indeed anxious, to devote the best years of their life to the study of medicine.

Medicine, indeed, needs no recruiting-sergeant, and has no necessity for any call to arms, for recruits are always ready, and notwithstanding the warnings given by the broken-down practitioners who abound on every side, and notwithstanding the obvious difficulty which is experienced by many who still keep upon their feet in making a respectable livelihood, we find a constant stream of new faces crowding forward to fill up, nay, even to overfill the ranks, and to carry on with renewed vigour that attack upon disease which is medicine's service to humanity. It is well, then, that we, who know somewhat of the inner life of the profession, and who know how hard that life in many cases is, should give a word of warning to those who in mere ignorance are rushing into a calling in which they are likely to meet with little but disappointment.

The first thing that we would insist upon is that, although medicine is a profession, and a noble one, success in it depends far more than many people imagine to be the case, upon the same careful and businesslike habits which lead to success in other careers which are looked upon as purely commercial. The next warning we would give is that the monetary reward for medical practice is very small. So much is heard of the leaders in the profession and so little note is taken of the position held by ordinary practitioners, that outsiders are apt to look upon medicine as a mine of wealth. But it is not. The wealth is for the few, the work is for the many; and although it is true enough that a steady man if blessed with good health is fairly sure of making a moderate living if he takes up general practice, much depends upon one's definition of the word "moderate." Something under £300 a year is, perhaps, the average, and a large number of very good men have, without doubt, to put up with a good deal less.

Nor is even that meagre income open to everyone who enters on the study of medicine. It is every year becoming more plain how severe is the weeding process which is exercised upon medical students by the modern examination system. The proportion who fail utterly and, after wasting their money and some of the best years of their lives, have to take to other kinds of work is very considerable. Still larger, however, is the number of those who, after entering in the hope of becoming qualified in five years, find that they have to take two or three shots at their examinations, and thus drag out their student life to a length which is absolutely ruinous to those on whom they depend for their support. This is a matter which should be very carefully borne in mind. No man can count upon getting through in the five years given in the prospectuses, and if he cuts his calculations fine he may come to the end of his resources long before he reaches his qualification, and thus all his money may be thrown away.

We cannot close without still another warning. Good health is an essential to success, whether as a student or as a practitioner. As a student, a few months' illness may knock a man out of his years, and leave him labelled as a "chronic" among men junior to himself, while as a practitioner it is notorious that a sharp illness, such as would do no harm to a commercial man's business, may absolutely ruin a promising practice. Nowhere is it more true than in medicine that the race is to the strong.

In view, then, of the fact that success in medicine requires business capacity, mental aptitude, good health, and a not inconsiderable amount of capital, we would ask those who are so endowed to consider carefully whether, if wealth and position is their aim, they could not get a better return for their talents in other fields?

